# Oral cancer: A multicenter study

**DOI:** 10.4317/medoral.21999

**Published:** 2017-12-24

**Authors:** Kittipong Dhanuthai, Somsri Rojanawatsirivej, Watcharaporn Thosaporn, Sompid Kintarak, Ajiravudh Subarnbhesaj, Mark Darling, Eugene Kryshtalskyj, Chun-Pin Chiang, Hong-In Shin, So-Young Choi, Sang-shin Lee, Pouyan-Amini Shakib

**Affiliations:** 1 Associate Professor, DDS, MSc, Department of oral Pathology, Faculty of Dentistry, Chulalongkorn University, Bangkok, Thailand; 2 Associate Professor, DDS, MSc, Department of Oral Biology and Oral Diagnostic Sciences, Faculty of Dentistry, Chiangmai University, Chiangmai, Thailand; 3 Assistant Professor, DDS, PhD, Department of Stomatology, Faculty of Dentistry, Prince of Songkla University, Songkhla, Thailand; 4 Assistant Professor, DDS, MSc, PhD, Department of Oral Diagnosis, faculty of Dentistry, Khon Kaen University, Khon Kaen, Thailand; 5 Associate Professor, BChD, MSc (Dent), MSc (Med), MChD, Department of Pathology and Laboratory Medicine, Western University, London, Canada; 6 DDS, BHSc (Hons), Department of Pathology and Laboratory Medicine Western University, London, Canada; 7 Professor, DDS, DMSC, Department of Oral Pathology and Oral Diagnosis, School of Dentistry, National Taiwan University, Taiwan; 8 Professor, DDS, PhD, Department of Oral Pathology, School of Dentistry, Kyungpook National University, Daegu, South Korea; 9 Assistant Professor, DDS, PhD, Department of Oral & Maxillofacial Surgery, School of Dentistry, Kyungpook National University, Daegu, South Korea; 10 Professor, DDS, PhD, Department of Oral Pathology, School of Dentistry, Gangneung-Wonju National University, Gangneung, South Korea; 11 Assistant Professor, DDS, MSc, Department of Oral and Maxillofacial Pathology, School of Dentistry, Tehran University of Medical Sciences, Tehran, Iran

## Abstract

**Background:**

To determine the prevalence and clinicopathologic features of the oral cancer patients.

**Material and Methods:**

Biopsy records of the participating institutions were reviewed for oral cancer cases diagnosed from 2005 to 2014. Demographic data and site of the lesions were collected. Sites of the lesion were subdivided into lip, tongue, floor of the mouth, gingiva, alveolar mucosa, palate, buccal/labial mucosa, maxilla and mandible. Oral cancer was subdivided into 7 categories: epithelial tumors, salivary gland tumors, hematologic tumors, bone tumors, mesenchymal tumors, odontogenic tumors, and others. Data were analyzed by descriptive statistics using SPSS software version 17.0.

**Results:**

Of the 474,851 accessioned cases, 6,151 cases (1.30%) were diagnosed in the category of oral cancer. The mean age of the patients was 58.37±15.77 years. A total of 4,238 cases (68.90%) were diagnosed in males, whereas 1911 cases (31.07%) were diagnosed in females. The male-to-female ratio was 2.22:1. The sites of predilection for oral cancer were tongue, labial/buccal mucosa, gingiva, palate, and alveolar mucosa, respectively. The three most common oral cancer in the descending order of frequency were squamous cell carcinoma, non-Hodgkin lymphoma and mucoepidermoid carcinoma.

**Conclusions:**

Although the prevalence of oral cancer is not high compared to other entities, oral cancer pose significant mortality and morbidity in the patients, especially when discovered late in the course of the disease. This study highlights some anatomical locations where oral cancers are frequently encountered. As a result, clinicians should pay attention to not only teeth, but oral mucosa especially in the high prevalence area as well since early detection of precancerous lesions or cancers in the early stage increase the chance of patient being cured and greatly reduce the mortality and morbidity. This study also shows some differences between pediatric and elderly oral cancer patients as well as between Asian and non-Asian oral cancer patients.

** Key words:**Oral cancer, prevalence, clinic-pathologic features, retrospective study.

## Introduction

Oral cancer is the sixth most common malignancy worldwide (1). Three hundred thousand patients (2.1% of the total cancer cases) were afflicted with cancer of the oral cavity and lip in 2012. One hundred and forty five thousand patients passed away from cancer of the oral cavity and lip (2).

It has long been accepted that tobacco consumption including smokeless tobacco and heavy alcohol consumption are the principal etiologic factors for the development of oral cancer. In addition, a variety of suspected risk factors such as chronic irritation, poor oral hygiene, viral infection, occupational exposure, malnutrition as well as low fruit and vegetable diets, and genetic factors, have been proposed for the development of oral cancer (3,4). The most important risk factors for squamous cell carcinoma are tobacco use and alcohol abuse, which have synergistic effect (5-7). Cigarette smoke contains more than 60 carcinogens according to the International Agency for Research on Cancer (8). Tobacco-specific N-nitrosamines, especially 4-(methylnitrosamino)-1-(3-pyridyl)-1-butanone (NNK) and N’-nitrosonornicotine (NNN), have been demonstrated to cause cancer in experimental animals. NNK is metabolically activated by cytochromes P450 to DNA-reactive metabolites which induce methylation as well as pyridyloxobutylation of nucleobases in DNA and form DNA adducts. For NNN, the 2’- and 5’-α-hydroxylation pathways are the major pathways leading to the formation of DNA adducts. The resulting DNA adducts may induce deleterious mutations in oncogenes and tumor suppressor genes which could be considered as tumor initiation (9,10). In the Indian subcontinent, some parts of Southeast Asia, and Taiwan, the use of betel quids containing areca nut and lime has long been strongly associated with an increased risk for oral cancer.

Previous studies have shown that alcohol consumption is an independent risk factor for the development of cancer in the dose dependent manner (11). Alcohol is first oxidized to acetaldehyde by alcohol dehydrogenase (ADH). Acetaldehyde is considered to be a group I carcinogen according to the International Agency for Research on Cancer (IARC). Acetaldehyde is further metabolized to acetate by aldehyde dehydrogenase (ALDH). Any defect in these enzymes (ADH and ALDH) may influence the carcinogenesis by alcohol (12). Alcohol also induces basal cell proliferation and generates free radicals which have the deleterious effects on DNA. In addition, alcohol-associated impairment of the body’s ability to breakdown and absorb nutrients and immune suppression may further promote carcinogenesis (6,7).

Apart from tobacco use and alcohol abuse, human papilloma virus has recently received special attention. Human papilloma virus, HPV-16 in particular, has been indicated as an etiological agent for the development of a subset of squamous cell carcinoma, especially at the base of the tongue and the tonsillar area in the younger individuals compared to the HPV-negative counterpart (13,14). The proportion of HPV-positive oropharyngeal cancer was 56% in North America, 52% in Japan, 45% in Australia, 39% in Northern and Western Europe, 38% in Eastern Europe, 17% in Southern Europe and 13% in the rest of the world (15).

There is a wide variation in the prevalence of oral cancer in different regions of the world or even within the same countries from the minorities or sub-populations. The aims of this research were to report the prevalence as well as clinicopathologic features of the oral cancer patients from different parts of Asia and Canada and to compare them with patients from other parts of the world.

## Material and Methods

The biopsy records of the Department of Oral Pathology, Chulalongkorn University, Gangneung-Wonju National University and Kyungpook National University, Department of Oral Biology and Diagnostic Sciences, Chiangmai University, Department of Oral Diagnosis, Khon Kaen University, Department of Stomatology, Prince of Songkla University, Department of Oral and Maxillofacial Pathology, Tehran University of Medical Sciences, Department of Pathology and Laboratory Medicine, Western University and Department of Oral Pathology and Oral Diagnosis, School of Dentistry, National Taiwan University were reviewed for oral cancer cases diagnosed from 2005 to 2014. Demographic data and site of the lesion were also collected. Sites of the lesion were subdivided into lip, tongue, floor of the mouth, gingiva, alveolar mucosa, palate, buccal/labial mucosa, maxilla and mandible. Oral cancer was subdivided into 7 categories: epithelial tumors, salivary gland tumors, hematologic tumors, bone tumors, mesenchymal tumors, odontogenic tumors, and others. Data collected were analyzed by appropriate statistics using SPSS Statistics for Windows, Version 17.0. Chicago: SPSS Inc. A *P* value less than .05 was considered to be statistically significant. This research was approved by the ethical committee of the Faculty of Dentistry, Chulalongkorn University (no. 90/2015).

## Results

**Figure 1:**
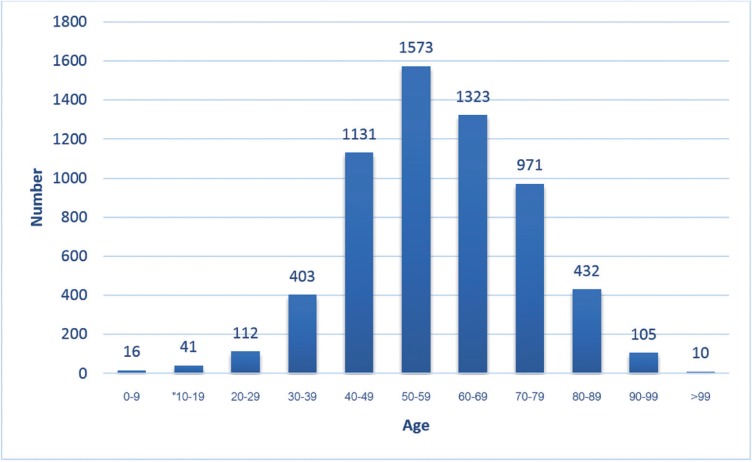
Age distribution of oral cancer patients.

Of the 474,851 accessioned cases, 6,151 cases (1.30%) were diagnosed in the category of oral cancer. The prevalence of oral cancer ranged from 0.83% in Taiwan to 6.23% in Thailand ( Table 1). The age of the patients in the present study ranged from 3 to 111 years with a mean ± SD = 58.37±15.77 years. The majority of the cases (81.26%) were encountered in the fifth to the eighth decades of life. Sixty seven cases (1.09%) were discovered in children aged 16 and below. Two thousand one hundred and forty eight cases (34.92%) were found in the elderly (aged 65 and above) (Fig. 1). Mean age of the pediatric patients±SD was 5.81±6.07, while that of the elderly patients was 75.0±7.74. Mean age of the Asian patients±SD was 56.37±14.98 years, while that of the non-Asian patients was 69.99±15.51 years. Mean age of the Asian patients was significantly lower than that of the non-Asian patients (*p*=0.000). Most countries demonstrated a male predilection except Thailand. The male-to-female ratio was 2.22:1. It was noteworthy that the male-to-female ratio from Taiwan was as high as 5.62:1 ( Table 1). Both the pediatric and elderly oral cancer patients elicited a slight male predilection with the male-to-female ratio of 1.31:1 and 1.28:1, respectively.

**Table 1:**
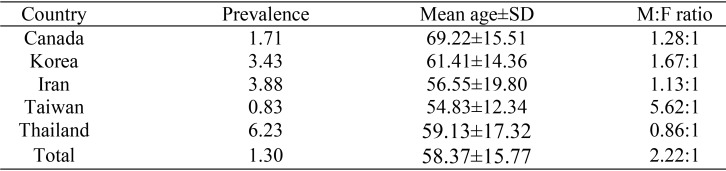
Prevalence, average age and M:F ratio of oral cancer patients.

Regarding the anatomical distribution of oral cancer, the 5 most common sites for oral cancer in descending order of frequency were tongue (25.4%), labial/buccal mucosa (21.7%), gingiva (14.0%), palate (9.9%), and alveolar mucosa (7.9%), respectively. Labial/buccal mucosa was the most common site for oral cancer in Taiwan, while tongue was the site of predilection for oral cancer in Canada and Iran and gingiva for Korea and Thailand. In children aged 16 and below, the 5 most common sites for oral cancer in descending order of frequency were mandible (17.9%), palate (14.9%), gingiva (13.4%), alveolar mucosa (11.9%), maxilla and tongue (10.4% each), respectively. In the elderly aged 65 and above, the 5 most common sites for oral cancer in descending order of frequency were tongue (20.8%), labial/buccal mucosa (18.2%), gingiva (16.0%), alveolar mucosa (14.5%), and palate (9.5%), respectively. Tongue was the site of predilection of oral cancer for both Asian and non-Asian patients. The second most common site for oral cancer in the Asian patients was labial/buccal mucosa, followed by gingiva and palate, respectively, while the second most common site for oral cancer in the non-Asian patients was alveolar mucosa followed by floor of the mouth and lip, respectively.

Most of the oral cancer (5,234 cases, 85.09%) fell in the epithelial tumor category followed by salivary gland tumor category (411 cases, 6.68%), hematologic tumor category (275 cases, 4.47%), bone tumor category (81 cases, 1.32%), mesenchymal tumor category (73 cases, 1.19%), others category (57 cases, 0.93%), and odontogenic tumor category (20 cases, 0.33%), respectively ( Table 2,  Table 2 continue). Epithelial tumor category ranked as the most common oral cancer category in all countries, while salivary gland tumor category came second in almost all countries except Canada and the third most common oral cancer category in most countries was hematologic tumor category except Canada. In Canada, the second most common oral cancer category was hematologic tumor category followed by salivary gland tumor category. The most common oral cancer was squamous cell carcinoma which constituted 94.08% of all epithelial tumors and 80.05% of all oral cancer cases. The second most prevalent oral cancer was lymphoma which accounted for 86.91% of the hematologic tumors and 3.89% of all oral cancer cases. The third most prevalent oral cancer was mucoepidermoid carcinoma which constituted 45.26% of all salivary gland tumors and 3.02% of all oral cancer cases. In the pediatric patients, squamous cell carcinoma was the most common tumor constituting 40.29% of the cases in this group, followed by mucoepidermoid carcinoma (16.42%) and lymphoma (14.93%), respectively. In the elderly patients, squamous cell carcinoma was the most prevalent tumor constituting 80.77% of the cases in this group, followed by verrucous carcinoma (5.42%), and lymphoma (5.21%), respectively. Squamous cell carcinoma was the most common oral cancer in both Asian (80.23%) and non-Asian patients (73.46%). The second most common oral cancer in Asian patients was verrucous carcinoma (3.37%) followed by lymphoma (3.33%), mucoepidermoid carcinoma (2.89%) and adenoid cystic carcinoma (1.85%), respectively, while the second most common oral cancer in non-Asian patients was lymphoma(6.90%) followed by mucoepidermoid carcinoma (3.76%), verrucous carcinoma (3.66%) and adenoid cystic carcinoma (2.19%), respectively. Thus the entities constituting the top five most common oral cancers for both Asian and non-Asian patients were exactly the same, but with different ranking.

**Table 2:**
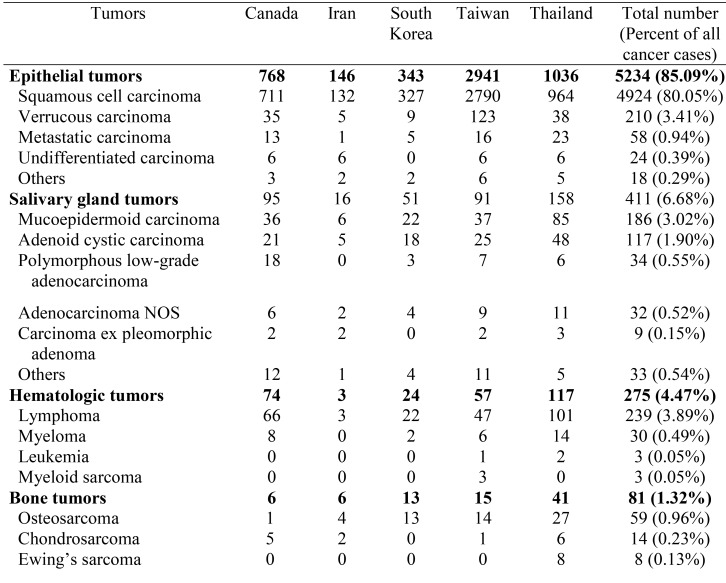
Histopathologic diagnosis of oral cancer patients.

**Table 2 continue:**
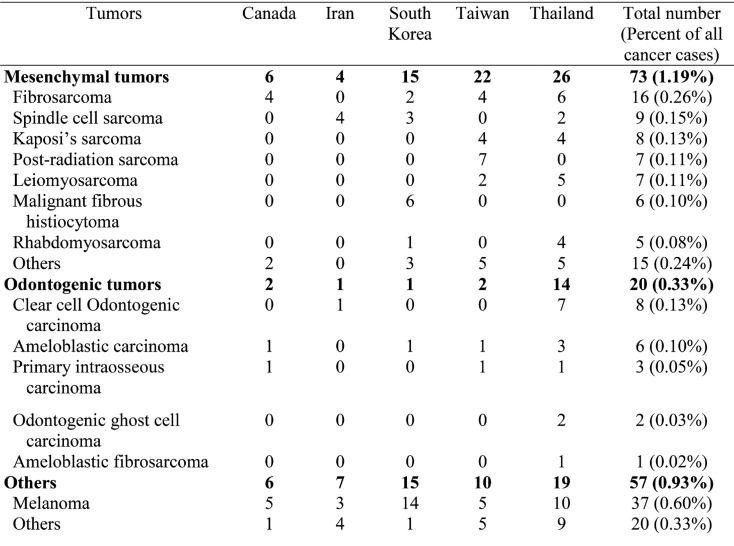
Histopathologic diagnosis of oral cancer patients.

There were 58 cases of metastatic tumors to the oral cavity which constituted 0.94% of the oral cancer. Mean age of the patients±SD was 61.31±16.23 years. Male-to-female ratio was 1.52:1. The sites of predilection for metastatic tumors were the mandible followed by the gingiva. Several sites such as lung, thyroid gland, breast, kidney liver, colon, pancreas, bile duct were the primary sites for the metastatic tumors to the oral cavity in the present study, but the most common ones were the thyroid gland and the lung.

## Discussion

The prevalence of oral cancer from the present study was 1.30% which was comparable to the prevalence of 0.15% from Australia (16), but lower than the prevalence of 6.23% from Thailand (17), 8.0% and 8.2% from Libya (18,19) 14.82% from UAE (20), 18.0% from Nigeria (21) and 24.8% from Zimbabwe (22). The disparity in the prevalence might be attributable to the difference in the distribution of risk factors in each geographical area (20,21). The mean age of the patients with oral cancer in this study was 58.37 years which is comparable to the findings in UAE (54.9 years) (20), Thailand (59.13 years) (17), Iran (61.2 years) (23), Malaysia (61.2 years) (24), Jordan (62.5 years) (25) and Japan (65.2 years) (26), but higher than the findings in Nigeria (42.2 years) (21), Libya (46.0 years) (18,19), and India (47.8 and 49.7 years) (27,28). Many more patients with oral cancer from this study were male with the male-to-female ratio of 2.22:1 which was in accordance with most previous reports which consistently showed a male predominance (18,19,22,23,25,26,29-32), but contrary to the Malaysian study which demonstrated the male-to-female ratio of 0.92:1 (24). Previous study of oral cancer from Thailand also reported more female than male patients as in the present study (17,33). As Malaysia and Thailand are neibouring countries, residents are exposed to similar type of environment and share some cultural practices, so both countries elicit a female predilection. In addition, more female patients seek medical attention than male patients in Thailand.

Most of the oral cancer in the present study were encountered at the tongue which was in accordance with most previous reports (20,23,26,30,32,34). The reasons why the tongue and the cheek are the predilection sites for oral cancer are that the carcinogens in the oral cavity mixing with saliva have the tendency to pool at the bottom of the mouth and these sites are covered by thin and non-keratinized mucosa. As a consequence, they provide less protection against the carcinogen (35). However, Chidzonza (22) reported that gingiva was the most common site for oral cancer followed by the tongue. Khan *et al.* (24) revealed that oral mucosa was the most common site for oral cancer followed by the tongue. Howell *et al.* (29) reported that the site of predilection for oral cancer was the lip followed by the tongue. The explanation for the high incidence of lip cancer is due to the overexposure to ultraviolet light, especially in Australia where residents have fair complexion. One study from India demonstrated that mandibular alveolus was the frequently involved site, followed by buccal mucosa. This is accounted for by the practice of betel quid/tobacco chewing (28). As a consequence, the alveolar mucosa, gingiva and buccal mucosa are constantly in contact with the carcinogens for a long period of time. This also explains the labial/buccal mucosa and gingiva as sites of predilection of oral cancer for Taiwan and Thailand among betel nut chewers.

Epithelial tumor category constituted the largest category of all the oral cancer followed by the salivary gland tumor category. This finding is in accordance with previous studies (18-24,32). Nonetheless, the studies by Ajayi *et al.* (21) and Rawashdeh & Matalka (25) revealed that sarcoma was the second most common category. Within the epithelial tumor category, squamous cell carcinoma was the most common tumor and the most common oral cancer as in previous studies (18-29,31,32). Within the salivary gland tumor category, the most prevalent tumor was mucoepidermoid carcinoma. Mucoepidermoid carcinoma was consistently ranked as the most common intraoral salivary gland tumor (19,20,24). However, some studies showed that adenoid cystic carcinoma was the most prevalent intraoral malignant salivary gland tumor (21,23).

In the present study, squamous cell carcinoma accounted for 80.05% of all oral cancer. This figure is lower than several previous studies which have reported that squamous cell carcinoma accounts for from 84.40% to 90% of all oral cancer (23-5,29,32,36). However, lower figures (63.00% to 73.10%) than the present study have been reported (20-22,34).

## Conclusions

Although the prevalence of oral cancer is not high compared to other entities, oral cancer pose significant mortality and morbidity in the patients, especially when discovered late in the course of the disease. This study highlights some anatomical locations where oral cancers are frequently encountered. As a result, clinicians should pay attention to not only teeth, but oral mucosa especially in the high prevalence area as well since early detection of precancerous lesions or cancers in the early stage increase the chance of patient being cured and greatly reduce the mortality and morbidity. This study also shows some differences between pediatric and elderly oral cancer patients as well as between Asian and non-Asian oral cancer patients.
